# Dental methacrylates may exert genotoxic effects via the oxidative induction of DNA double strand breaks and the inhibition of their repair

**DOI:** 10.1007/s11033-012-1582-3

**Published:** 2012-02-12

**Authors:** Janusz Blasiak, Ewelina Synowiec, Justyna Tarnawska, Piotr Czarny, Tomasz Poplawski, Russel J. Reiter

**Affiliations:** 1 Department of Molecular Genetics, University of Lodz, Pomorska 141/143, 90-236 Lodz, Poland; 2 Department of Cellular & Structural Biology, The University of Texas Health Science Center, 7703 Floyd Curl Drive, San Antonio, TX 78229-3900 USA

**Keywords:** Methacrylate-based dental materials, DNA damage, DNA repair, DNA double-strand breaks, 2-hydroxyethyl methacrylate, HEMA, Bisphenol A-diglycidyl dimethacrylate, Bis-GMA, Vitamin C, Melatonin

## Abstract

Methacrylate monomers used in dentistry have been shown to induce DNA double strand breaks (DSBs), one of the most serious DNA damage. In the present work we show that a model dental adhesive consisting of 45% 2-hydroxyethyl methacrylate (HEMA) and 55% bisphenol A-diglycidyl dimethacrylate (Bis-GMA) at concentrations up to 0.25 mM Bis-GMA induced oxidative DNA in cultured primary human gingival fibroblasts (HGFs) as evaluated by the comet assay and probed with human 8-hydroxyguanine DNA-glycosylase 1. HEMA/Bis-GMA induced DSBs in HGFs as assessed by the neutral comet assay and phosphorylation of the H2AX histone and sodium ascorbate or melatonin (5-methoxy-*N*-acetyltryptamine) both at 50 μM reduced the DSBs, they also inhibited apoptosis induced by HEMA/Bis-GMA. The adhesive slowed the kinetics of the repair of DNA damage induced by hydrogen peroxide in HGFs, while sodium ascorbate or melatonin improved the efficacy of H_2_O_2_-induced damage in the presence of the methacrylates. The adhesive induced a rise in the G2/M cell population, accompanied by a reduction in the S cell population and an increase in G0/G1 cell population. Sodium ascorbate or melatonin elevated the S population and reduced the G2/M population. In conclusion, HEMA/Bis-GMA induce DSBs through, at least in part, oxidative mechanisms, and these compounds may interfere with DSBs repair. Vitamin C or melatonin may reduce the detrimental effects induced by methacrylates applied in dentistry.

## Introduction

Methacrylate monomers are used in the restorative and aesthetic dentistry to produce polymers displaying excellent mechanical properties and a high affinity to the tooth enamel and dentin. However, the process of polymerization of monomers, which is lead in situ, is never complete, resulting in the release of free monomers into the oral cavity [1]. The monomers can be also released, along with other low-molecular weight molecules, as a consequence of the degradation of polymers by enzymes present in the saliva and mechanical shearing associated with chewing [2]. Methacrylate monomers may migrate to the pulp through microtubules present in the dentin. Therefore, they can reach the bloodstream and virtually all organs. The possible local concentration of released methacrylates was estimated to be high enough to induce adverse biological effects [3].

Cytotoxicity and genotoxicity of methacrylate monomers used in dentistry were reported in several studies [4]. Their ability to interact directly with DNA was repeatedly confirmed [4–12]. Recently, some methacrylate monomers, most commonly used in dentistry, were reported to induce DNA double-strand breaks (DSBs) in human gingival fibroblasts (HGFs) [13]. DSBs are one of the most serious DNA damage, which may have severe phenotypic consequences if not repaired [14]. Due to common use of methacrylate-based dental restorations, preventive and protective actions against DSBs induced by methacrylate monomers should be investigated. In our recent work we showed that chitosan oligosacccharide lactate, a chitosan derivative, may protect HGFs against DSBs induced by methacrylate monomers: 2-hydroxyethyl methacrylate (HEMA) and bisphenol A-diglycidyl dimethacrylate (Bis-GMA) [15]. However, the formulation of methacrylates with the chitosan derivative, ensuring its protective action in the target sites, has not been established. A better understanding of mechanisms leading to the induction of DSBs by methacrylate monomers may help in the planning of a strategy to reduce the detrimental impact of these substances.

Methacrylate monomers were reported to induce oxidative DNA damage. An important role of such damage in the toxicity of dental methacrylates was confirmed recently by Schweikl and coworkers [16], who directly showed the production of reactive oxygen species (ROS) by triethylene glycol dimethacrylate (TEGDMA) in human fibroblasts. Oxidative mechanisms can likely contribute to the ability of methacrylates to induce DSBs [17].

A genotoxic effect induced by a chemical substance in a cell may be expressed by the net extent of DNA damage. Many factors influence this extent, first of all the cell’s basic metabolism, including its ability to detoxify chemicals with enzymes and low-molecular weight protective substances, regulation of the cell cycle and DNA repair, which are elements of the DNA damage response [18]. However, in a particular cellular condition, the extent of DNA damage induced by a chemical depends mainly on its ability to induce DNA damage per se and its influence on the process of repair of such damage.

In the present work, we investigated the role of oxidative mechanisms in the ability of the mixture of HEMA and Bis-GMA monomers to induce DSBs and the potential of the monomers to interfere with DNA repair in HGFs. The ability of the methacrylates to cause oxidative DNA damage was assessed by the quantification of 8-oxo-7,8-dihydroguanine (8-oxoGua), which is frequently used as a biomarker of oxidative DNA damage [19], by the enzyme human 8-hydroxyguanine DNA-glycosylase 1 (hOGG1), which recognizes modified bases and removing them. We used commonly recognized antioxidants, sodium ascorbate, a form of vitamin C and melatonin to assess the role of oxidative mechanisms in the induction of DSBs by the methacrylate monomers.

## Materials and methods

### Chemicals

HEMA (CAS 868-77-9), Bis-GMA (CAS 1565-94-2), gradisol and RNase A, low melting point (LMP) and normal melting point (NMP) agarose, phosphate buffered saline (PBS), DAPI (4′,6-diamidino-2-phenylindole), dimethyl sulfoxide (DMSO), fetal bovine serum (FBS), MTT, lectin, penicillin, streptomycin, sodium ascorbate, Bradford reagent were from Sigma Chemicals (St. Loius, MO, USA). hOGG1 was purchased from New England Biolabs (Herts, UK). Melatonin (5-methoxy-*N*-acetyltryptamine) was provided by R.J. Reiter of University of Texas Health Science Center. Quantum 333 medium, Dulbecco’s phosphate buffered saline (DPBS), trypsin and EDTA were from PAA Laboratories GmbH (Cölbe, Germany). Methanol-free formaldehyde solution was from Thermo Fisher Scientific, Worcester, MA, USA. Mouse monoclonal anti-γ-H2AX primary antibody, 1:100 dilution, anti-phospho-histone H2A.X (Ser139) clone JBW301, was obtained from Upstate (Charlotesville, VA, USA). Alexa Fluor 488 secondary antibody, 1:100 dilution, conjugated goat anti-mouse IgG was from Molecular Probes (Eugene, OR, USA). Cell viability kit was purchased in BD Biosciences (San Jose, CA, USA). All other chemicals were of the highest commercial grade available.

### Cells and treatment

HGFs cell line was purchased from Provitro (Berlin, Germany). The cells were grown in Quantum 333 medium containing l-glutamine and supplemented with 1% antibiotic–antimycotic solution (10,000 U/ml penicillin, 10 mg/ml streptomycin sulphate, 25 μg/ml amphotericin B) in 75 cm^2^ cell culture flasks to approximately 75–80% confluence and maintained in an incubator with 5% CO_2_ atmosphere at 100% humidity at 37°C. After reaching confluence, the cells were washed with DPBS, detached from the flasks by a brief treatment with 0.05% trypsin-0.02% EDTA.

The model adhesive consisted of HEMA and Bis-GMA at 45/55% w/w with 8% water based on the total final weight of the mixture [20]. To obtain a well-mixed resin we applied extensive shaking and sonication. The mixture was diluted with the cells medium to the concentrations desired in the experiments on DNA damage. HGFs were exposed to HEMA/Bis-GMA mixture at appropriate concentrations for 6 h at 37°C. We incubate HGFs with HEMA/Bis-GMA mixture, with reference to Bis/GMA at 0.01, 0.25, 0.05, 0.1 and 0.2 mM in DNA damage experiments and at 0.1 and 1.0 mM in apoptosis and cell cycle experiments. In the experiment with antioxidants, the exposure to HEMA/Bis-GMA was preceded by an 1 h incubation with sodium ascorbate or melatonin at 37°C. After the incubation, the suspension of the cells was centrifuged to remove free antioxidants. Each DNA damage experiment included a positive control, which was hydrogen peroxide at 20 μM for 15 min on ice [21]. In the H2AX histone phosphorylation experiment the concentration of hydrogen peroxide was 1 mM.

### Assessment of oxidative DNA damage

The human hOGG1, the primary enzyme for the repair of 8-oxoGua, was used to assess the extent of oxidative modification to the DNA bases [22, 23]. The enzyme nicks the DNA strand at the 8-oxoGua sites, producing single-strand breaks (SSBs) which can be easily detected by the alkaline comet assay.

The comet assay was performed under alkaline conditions essentially according to the procedure of Singh et al. [24] with modifications [25] as described previously [26]. A freshly prepared suspension of cells in 0.75% LMP agarose dissolved in PBS was spread onto microscope slides precoated with 0.5% NMP agarose. The cells were then lysed for 1 h at 4°C in a buffer consisting of 2.5 M NaCl, 100 mM EDTA, 1% Triton X-100, 10 mM Tris, pH 10. After lysis, the slides were placed in an electrophoresis unit, the DNA was allowed to unwind for 20 min in the electrophoretic solution consisting of 300 mM NaOH, 1 mM EDTA, pH >13. Electrophoresis was conducted at 4°C (the temperature of the running buffer did not exceed 12°C) for 20 min at an electric field strength of 0.73 V/cm (290 mA).

The slides were then washed in water, drained and stained with 2 μg/ml DAPI and covered with cover slips. To prevent additional DNA damage, all the steps described above were conducted under dimmed light or in the dark. The slides were observed at ×200 magnification using an Eclipse fluorescence microscope (Nikon, Tokyo, Japan) attached to a COHU 4910 video camera (Cohu, Inc., San Diego, CA, USA) equipped with a UV-1 filter block consisting of an excitation filter (359 nm) and barrier filter (461 nm) and connected to a personal-computer-based image analysis system, Lucia-Comet v. 4.51 (Laboratory Imaging, Praha, Czech Republic). A hundred images was randomly selected from each sample and the comet tail DNA (% tail DNA) was measured. Each experiment was repeated three times. % tail DNA is positively correlated with the level of DNA breakage or/and alkali labile sites and is negatively correlated with the level of DNA crosslinks in the alkaline version of the comet assay [17]. In the pH 12.1 and neutral version, it is positively correlated with strand breaks and DSBs, respectively. The mean value of the % tail DNA in a particular sample was taken as an index of the DNA damage in this sample.

After incubation with HEMA/Bis-GMA and cell lysis the slides from the comet assay were washed three times in the enzyme buffer containing 40 mM HEPES–KOH, 0.1 M KCl, 0.5 mM EDTA, 0.2 mg/ml bovine serum albumin, pH 8.0 for 5 min each time and drained. The agarose on slides was covered with 30 μl of the enzyme buffer either with or without hOGG1 at 1 μg/ml, sealed with a cover glass and incubated for 10 min at 37°C [27]. The slides were processed as described in “Cells and treatment” section. To check the ability of the enzyme to recognize the DNA oxidative damage, we exposed HGF to 20 μM hydrogen peroxide for 10 min on ice (positive control). We compared the values obtained for the hOGG1 enzyme with the control containing only enzyme buffer.

### DNA DSBs assay

The neutral comet assay was used to screen for DSBs in HGFs [28]. In this version of the assay electrophoresis was run in a buffer consisting of 100 mM Tris and 300 mM sodium acetate at pH adjusted to 9.0 by glacial acetic acid. Electrophoresis was conducted for 60 min, after a 20 min equilibrium period, at electric field strength of 0.41 V/cm (50 mA) at 4°C. The slides were then proceeded as described in “Assessment of oxidative DNA damage” section. In this version, the mean value of the % tail DNA in a particular sample was taken as an index of the DNA DSBs in this sample.

The ability of the methacrylate monomers of the HEMA/Bis-GMA model adhesive to induce DSBs was confirmed and further analyzed by the immunofluorescence assay for the phosphorylation of the H2AX histone [29]. HGFs were grown to approximately 75–80% confluence in 6-well plates. The medium was changed 24 h before incubation with the mixture with at 100 μM Bis-GMA. After the incubation, the cells were trypsinized with 500 μl trypsin–EDTA, washed with 1 ml medium and collected in 1.5 ml tubes. For immunofluorescent staining, cells (1–2 × 10^6^) were washed in DPBS by centrifugation (300×*g* for 5 min at room temperature), fixed by 1 ml ice-cold 1% methanol-free formaldehyde in DPBS and incubated on ice for 15 min. Cells were centrifuged (300×*g*, 5 min, room temperature) and permeabilized with 80% ethanol in distilled water and kept at −20°C for 2 h until further staining. Cells were then washed three times with 1% BSA/0.2% Triton X-100/PBS (BTP) solution and stained with mouse monoclonal anti γ-H2AX primary antibody and incubated overnight at 4°C. Then, HGFs were washed three times with BTP solution and incubated with Alexa Fluor 488 secondary antibody for 1 h at room temperature in the dark. After the incubation cells were washed in BTP and counterstained with propidium iodide (PI, 5 μg/ml in DPBS in the presence of 100 μg/ml of RNase A) and incubated for 30 min at room temperature in the dark. Cells stained with Alexa Fluor 488 and PI were analyzed with LSRII flow cytometer (Becton–Dickinson Biologicals, San Jose, CA, USA) by measuring the intensity of green (530 ± 20 nm) and red (>600 nm) fluorescence of the cells. DNA content (red fluorescence of DNA-bound PI) was plotted on the x-axis and the level of γ-H2AX immunofluorescence (green fluorescence—Alexa Fluor 488) was plotted on the y-axis. Logarithmic Alexa Fluor 488 fluorescence was plotted versus linear PI fluorescence using FlowJo analysis software (TreeStar, Ashland, OR, USA). Untreated controls were used to set the threshold gating to determine the percentage of γ-H2AX positive cells. Intensity of cellular γ-H2AX immunofluorescence measured by flow cytometry is positively correlated with the level of DSBs and was used to quantify their extent [30, 31].

### DNA repair

To examine DNA repair, cells after a 10 min pre-treatment with hydrogen peroxide at 100 μM on ice were washed and resuspended in a fresh medium containing HEMA/Bis-GMA at 10 μM Bis-GMA preheated to 37°C. Aliquots of the suspension were taken immediately and 30, 60, 90, and 120 min later. Placing the samples in an ice bath stopped the repair activity of cells. The kinetics of DNA repair was quantified by determination the extent of residual DNA damage at each time-point with using the comet assay.

### Apoptosis

The BD Annexin V-FITC Apoptosis Detection Kit I was used to measure apoptosis. The kit contains Annexin V conjugated to the flurochrome FITC that has affinity for phosphatidylserine, which is transferred through cell membrane in the earlier stages of apoptosis. Propidium iodine was used to distinguish early apoptotic cells from cells undergoing late apoptosis or necrosis. Cells that are viable are Annexin V-FITC and PI negative, cells that are in early apoptosis are Annexin-FITC positive and PI negative, cells that are in late apoptosis are both Annexin-FITC and PI positive, cells already dead are only PI positive. After 6 h of incubation with HEMA/Bis-GMA, cells were washed in cold medium and resuspended in 1× binding buffer at 10^6^ cells/ml. 5 μl of Annexin V-FITC and 5 μl of PI were added to an aliquot of 100 μl (10^5^ cells) of cells suspension, gently mixed by pipetting and incubated for 30 min at room temperature in the dark. Next, 400 μl of 1× binding buffer was added to each tube and samples were analyzed by flow cytometry. Each experiment had a negative, positive and unstained control sample. About 10,000 events were counted per sample. The apoptosis ratio was calculated as a percent of apoptotic cells in a sample.

### Cell cycle

The CycleTEST PLUS DNA Reagent Kit was used to determine the DNA index (DI) and cell-cycle phase distributions. Nuclei were isolated, stained with propidium iodine and afterward analyzed on the LSRII flow cytometer according to the manufacturer instruction. The DI was calculated by dividing the mean of the relative content of the exposed G0/G1 population by the mean of the control G0/G1 population. Results were analyzed by FlowJo software, v. 7.2.4.

### Data analysis

The values in this study were expressed as mean ± SEM from three experiments, i.e. the data from three experiments were pooled and the statistical parameters were calculated. The Mann–Whitney test was used to determine differences between samples with distributions departing from normality. The differences between samples with the normal distribution were evaluated by applying the Student’s *t* test. Data analysis was performed using SigmaStat software (v. 3.0.0, SPSS, Chicago, USA).

## Results

### HEMA/Bis-GMA induces oxidative DNA damage

**Fig. 1:**
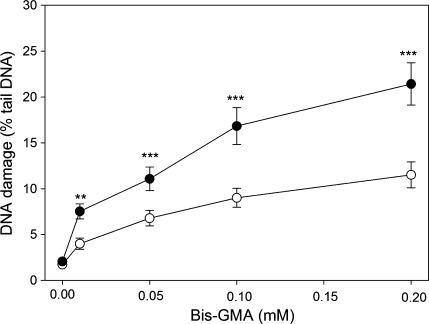
Oxidative DNA base modifications evoked by a mixture of methacrylates containing 45% 2-hydroxyethyl methacrylate and 55% bisphenol A-diglycidyl dimethacrylate (w/w) (HEMA/Bis-GMA) during a 6 h exposure at 37°C without (*empty symbols*) or with (*filled symbols*) a subsequent 15-min incubation with human 8-hydroxyguanine 1 (hOGG1) at 1 μM. The base modifications were measured as percentage of DNA in the tail in the alkaline comet assay. The concentration of the mixture is expressed as the Bis-GMA concentration. The number of cells analyzed for each sample was 100. The results are the mean of three independent experiments. *Error bars* denote SEM, **p < 0.01, ***p < 0.001 as compared with the unexposed to the mixture controls

Figure 1 presents the mean % tail DNA of HGFs exposed for 6 h at 37°C to HEMA/Bis-GMA mixture, lysed and post-treated with hOGG1, reduced by mean % tail DNA for cells incubated only with enzymatic buffer. For all HEMA/Bis-GMA concentrations, DNA damage observed in HGFs treated with the enzyme was significantly greater than the damage for untreated cells. This result indicates that oxidative modifications to the DNA bases play a role in the DNA-damaging action of HEMA/Bis-GMA. The difference between the ordinates of the points of two curves represent a net number of oxidatively modified bases, which were not recognized in the comet assay without hOGG1.

### HEMA/Bis-GMA induces DNA DSBs and sodium ascorbate or melatonin decrease their extent

**Fig. 2:**
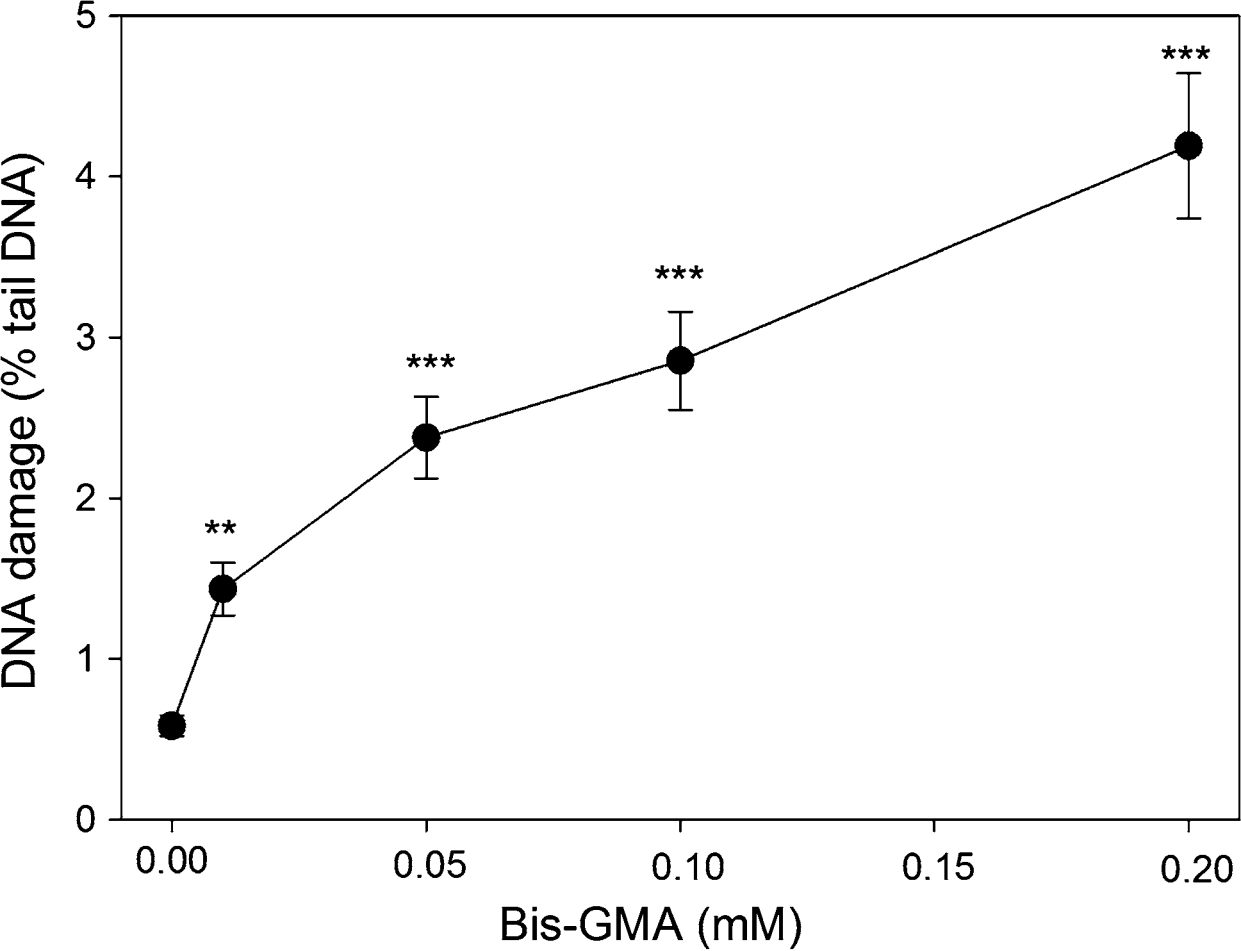
DNA damage in human gingival fibroblasts exposed for 6 h at 37°C to the mixture of methacrylates containing 45% 2-hydroxyethyl methacrylate and 55% bisphenol A-diglycidyl dimethacrylate (w/w) (HEMA/Bis-GMA) at different Bis-GMA concentrations. DNA damage was measured as percentage in the tail DNA in comets using the neutral version of the comet assay. The mean value for one hundred cells analyzed at each concentration in three independent experiments is displayed; *error bars* represent SEM, **p < 0.01, ***p < 0.001 as compared with unexposed controls

**Fig. 3:**
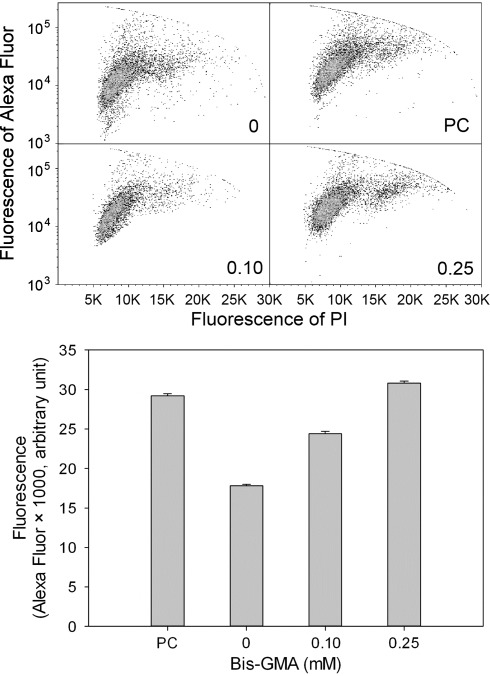
DNA DSBs in human gingival fibroblasts exposed for 6 h at 37°C to the mixture of methacrylates containing 45% 2-hydroxyethyl methacrylate and 55% bisphenol A-diglycidyl dimethacrylate (w/w) (HEMA/Bis-GMA) at different Bis-GMA concentrations evaluated by the phosphorylation of the H2AX histone assay and compared with unexposed controls. The intensity of fluorescence of the phosphorylated histone, γ-H2AX, is plotted and this quantity is positively correlated with the number of DSBs. Hydrogen peroxide was used as a positive control (PC). The cells were incubated with appropriate antibodies, stained with Alexa Fluor and propidium iodine and analyzed by flow cytometry (*upper diagrams*, numbers in the *lower*-*right* *corner* correspond to HEMA/Bis-GMA concentration in mM). *Error bars* denote SEM, *p* < 0.001 in all cases

The results of neutral comet assay suggest that HEMA/Bis-GMA might induce DSBs in HGFs (Fig. 2). We verified this hypothesis in the phosphorylation of H2AX histone test. The results (Fig. 3) confirmed the ability of the mixture to induce DSBs. Again, we observed a concentration-dependent rise in the extent of DSBs and the highest HEMA/Bis-GMA, 0.25 mM Bis-GMA, almost doubled the number of DSBs observed in the control (*p* < 0.001 for all concentrations). Therefore, the suitability of the neutral comet assay to detect DSBs induced by HEMA/Bis-GMA was confirmed in these experimental conditions so it was used in subsequent experiments to assess the influence of antioxidants and DNA repair.

**Fig. 4:**
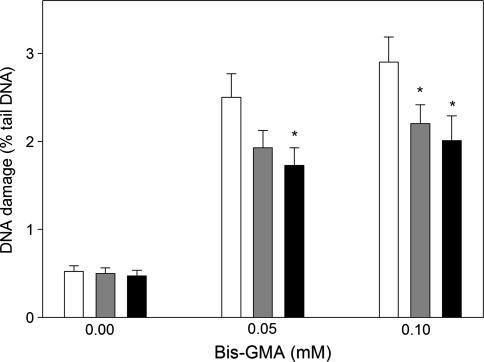
DNA damage in human gingival fibroblasts exposed for 6 h at 37°C to the mixture of methacrylates containing 45% 2-hydroxyethyl methacrylate and 55% bisphenol A-diglycidyl dimethacrylate (w/w) (HEMA/Bis-GMA) at different on Bis-GMA concentrations without (*white bars*) and with a 1 h preincubation with 50 μM ascorbate (*grey bars*) or 50 μM melatonin (*black bars*). DNA damage was measured as percentage in the tail DNA in comets using the neutral version of the comet assay. The mean value for one hundred cells analyzed at each concentration in three independent experiments is displayed; *error bars* represent SEM, *p < 0.05 as compared with samples without preincubation

Preincubation with sodium ascorbate and melatonin, both at 50 μM, decreased the extent of DSBs induced by HEMA/Bis-GMA (Fig. 4). Although the decrease evoked by sodium ascorbate at 0.05 mM Bis-GMA was not statistically significant (*p* = 0.085) we think that it should be taken into account, especially that the ascorbate significantly decreased the extent of DSBs at 0.1 Mm Bis-GMA. The decrease depended on the concentration of Bis-GMA and ranged from 22 to 31%. Therefore, we considered it as biologically significant.

### HEMA/Bis-GMA may influence the kinetics of DSBs repair

**Fig. 5:**
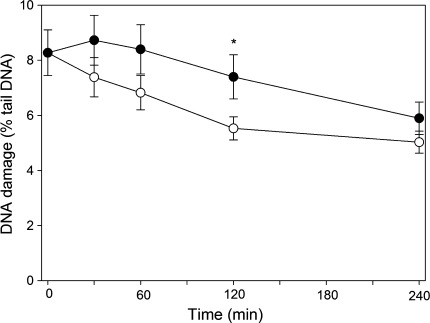
Time-course of DNA DSB repair in human gingival fibroblasts exposed for 10 min to hydrogen peroxide at 100 μM on ice. After the exposure, the cells were washed and divided into two aliquots, one of which was incubated at 37°C in the absence (*empty symbols*), and the other in the presence of the mixture of methacrylates containing 45% 2-hydroxyethyl methacrylate and 55% bisphenol A-diglycidyl dimethacrylate (w/w) (HEMA/Bis-GMA) (*filled symbols*) at 10 μM Bis-GMA. The repair was assessed as the decrease in the extent of DNA damage measured at indicated time as the percentage in the tail DNA in comets using the neutral version of the comet assay. The number of cells analyzed in each time-interval was 100. The results are mean of three independent experiments. *Error bars* denote SEM, *p < 0.05 as compared with the extent of DNA damage in incubation in methacrylate-free medium

In this experiment, HGFs were challenged by a high concentration of H_2_O_2_, which was washed out and the kinetics of DNA repair was measured in the presence of 10 μM HEMA/Bis-GMA. We measured the percentage of DNA in the comet tail immediately after the exposure to H_2_O_2_ (time “zero”) and after additional 30, 60, 120 and 240 min (Fig. 5). We observed a weak kinetics of removing DSBs induced by H_2_O_2_. During a 2-h repair incubation the extent of DNA damage decreased about one-fourth. In the case of the cells exposed to H_2_O_2_, they were post-incubated in a methacrylate-free medium, so they might repair the damage to their DNA. In the cells, which were post-incubated with HEMA/Bis-GMA mixture, two processes could occur: DNA repair and the induction of DNA damage. The repair might involve removal of damage introduced during pre-incubation with H_2_O_2_ and the repair of damage induced by the methacrylates during post-incubation. The induction of DNA damage could be noted after 60 min of repair incubation, although the increase in tail DNA observed then was not statistically significant. The cells incubated with HEMA/Bis-GMA apparently recovered more slowly from the DNA damage induced by H_2_O_2_ up to at least 120 min after the challenge with a statistically significant (*p* < 0.05) difference at 120 min of the repair incubation.

### Sodium ascorbate or melatonin protect HGFs from apoptosis induced by HEMA/Bis-GMA

**Fig. 6:**
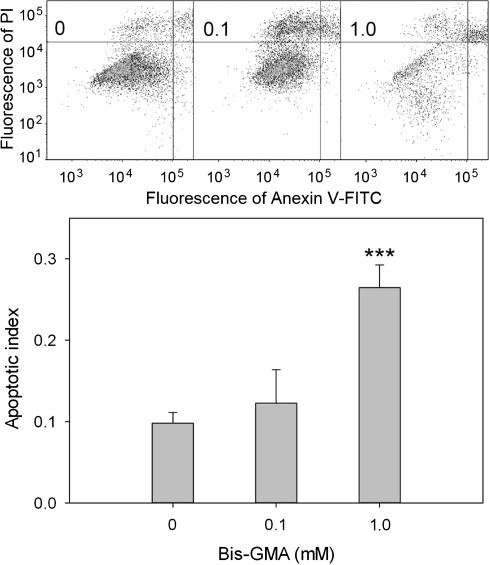
Apoptosis of human gingival fibroblasts exposed for 6 h at 37°C to the mixture of methacrylates containing 45% 2-hydroxyethyl methacrylate and 55% bisphenol A-diglycidyl dimethacrylate (w/w) (HEMA/Bis-GMA). Apoptosis was assessed by flow cytometry with Annexin V-FITC/propidium iodine (PI). Displayed is the mean of three experiments of 5 × 10^4^ measurements each; *error bars* denote SEM. The contour diagrams above the plot show the results of one representative experiment out of three for each HEMA/Bis-GMA concentration. The *lower left quadrant* of each diagram show the viable cells, which exclude PI and are negative for Annexin V-FITC binding. The *upper right quadrants* contain the non-viable, necrotic cells, positive for Annexin V-FITC binding and for PI uptake. *The lower left quadrants* represent the apoptotic cells, Annexin V-FITC positive and PI negative, demonstrating cytoplasmic membrane integrity. Apoptotic index was calculated as a ratio of the number of early and late apoptotic cells to the number of cells with no evidence of apoptosis; ***p < 0.001 as compared with unexposed control

**Fig. 7:**
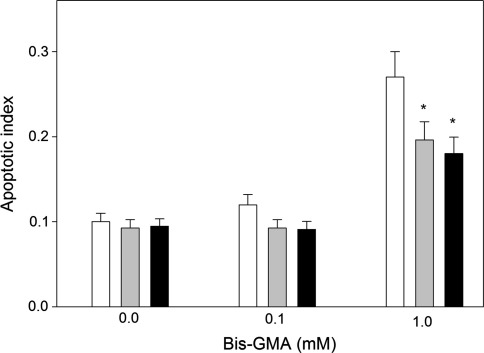
Apoptosis of human gingival fibroblasts exposed for 6 h at 37°C to the mixture of methacrylates containing 45% 2-hydroxyethyl methacrylate and 55% bisphenol A-diglycidyl dimethacrylate (w/w) (HEMA/Bis-GMA) at different Bis-GMA concentration without (*white bars*) or with a 1-hr incubation with 50 μM ascorbate (*grey bars*) or melatonin at the same concentration (*black bars*). Apoptosis was assessed by flow cytometry with Annexin V-FITC/propidium iodine. Apoptosis was expressed as a ratio of the number of early and late apoptotic cells to the number of cells with no evidence of apoptosis; *p < 0.05 as compared with unexposed control

HEMA/Bis-GMA induced apoptosis in HGFs after a 6-h incubation in a concentration-dependent manner (Fig. 6). At 1.0 mM HEMA/Bis-GMA the percentage of apoptotic cells was almost three times greater than in the control. A 1-h preincubation with 50 μM sodium ascorbate or melatonin reduced the number of apoptotic cells (Fig. 7). At 1 mM this reduction was significant (*p* < 0.05) and was about 27% for the ascorbate and 33% for melatonin.

### HEMA/Bis-GMA induce changes in the cell cycle and these changes are modulated by sodium ascorbate or melatonin

**Fig. 8:**
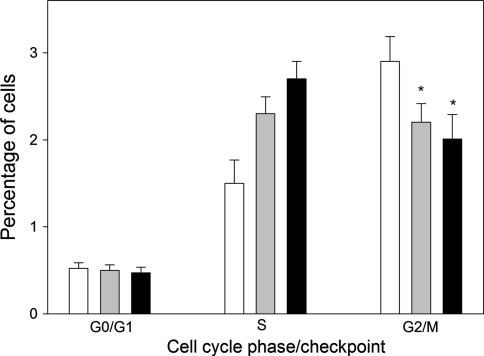
Cell cycle analysis in human gingival fibroblasts exposed for 6 h at 37°C to the mixture of methacrylates containing of 45% 2-hydroxyethyl methacrylate and 55% bisphenol A-diglycidyl dimethacrylate (w/w) (HEMA/Bis-GMA) for 6 h at 37°C in dependence on Bis-GMA concentration without (*white bars*) or with a 1-hr incubation with 50 μM ascorbate (*grey bars*) or melatonin at the same concentration (*black bars*). Percentage of cells in the G0/G1, S and G2/M stage of the cell cycle after treatment with HEMA/Bis-GMA is presented along with histograms for each HEMA/Bis-GMA concentration. Nocodazole (Noc) was used as a positive control. Data are expressed as means of three independent experiments; *error bars* denote SEM, *p < 0.05 as compared with unexposed control. The Bis-GMA axis is not linear

To determine the effect of HEMA/Bis-GMA on the HGFs cell cycle progression, the cells were exposed to increasing concentrations of the mixture for 24 h. Immediately after the exposure to HEMA/Bis-GMA, the cells were stained (0.5 h) and analyzed with FACS. HEMA/Bis-GMA evoked cell cycle arrest at the G2/M checkpoint (Fig. 8). The increase in the G0/G1 cell population was combined with the decrease in the S as well as G2/M cell population.

Sodium ascorbate or melatonin changed the pattern of cell distribution according to their cycle phase (Fig. 8). Both substances increased the S population and decreased the G2/M population.

## Discussion

We confirmed the results obtained by Urcan et al. [13] that methacrylate monomers used in dentistry may induce DSBs. Those authors used probably the most reliable method to detect and quantify DSBs—the H2AX histone phosphorylation test. We also used that test, additionally, we obtained a positive result from the neutral comet assay. The neutral version of the comet assay may be less reliable than the H2AX assay for assessing the presence or extent of DSBs because DNA SSBs may interfere with measuring the breaks in this method. This interference occurs because the relaxation of DNA supercoils, which is essential for the image of a comet tail, may occur at both neutral and alkaline pH.

We examined another possible pathway of the mechanism of genotoxicity of dental methacrylates—interference with DNA repair. We investigated this possibility in the case of DSBs repair. We obtained results suggesting that HEMA/Bis-GMA might inhibit DSBs repair, but these results are not completely clear. As mentioned above, when the influence of a chemical on the kinetics of DNA repair on the basis of the decrease in the extent of DNA damage was investigated, we should take into account that the observed extent of DNA damage is a result of the interaction of methacrylate with DNA repair processes and its ability to induce DNA damage. Therefore, we concluded that HEMA/Bis-GMA may interact with DSBs repair and this should be further investigated. DSBs in human cells are primarily repaired by a relatively fast, but sometimes error-prone non-homologous end joining (NHEJ) with its backup variant (B-NHEJ), and slower, but accurate homologous recombination repair (HRR) [32]. On the basis of the results we obtained it is not possible to determine which pathway may be affected by methacrylates since both DNA repair systems are multi-stage and multi-protein pathways.

We show herein that oxidative mechanisms likely underline, at least in part, the observed DNA-damaging potential of methacrylate monomers. Oxidative aspects of the genotoxicity of dental materials were shown in a series of elegant papers of Schweikl and his co-workers [7, 33]. We also reported previously that DNA damage induced by several methacrylates might include modifications of DNA bases, which are recognized by DNA repair enzymes preferentially targeting oxidative DNA modifications [8–11]. In the present work we also documented that the ability of methacrylates to induce DSBs, the most serious DNA damage, may be underlined by oxidative mechanisms. This suggests that recognized antioxidants may be useful in the prevention of the genotoxic action of methacrylate monomers. One such substances is melatonin, an indeloamine produced in the pineal gland and other organs [34]. Recently we showed that melatonin reduces the genotoxic effects of HEMA/Bis-GMA but does not interfere with mechanical properties of HEMA/Bis-GMA-based dental fillings [35].

DNA repair is the primary response of cells to DNA damage and if the cell cannot cope with DNA damage it may activate a checkpoint, G2/M or G1/S, which induces cell cycle arrest. This allows more time for DNA repair. If the cell cannot complete the repair of its DNA, it may enter the apoptotic pathway, saving itself from cancer transformation. We observed here and elsewhere the cell cycle changes in the presence of methacrylates and such changes were also reported by others [7, 33, 36, 37]. The changes in the HGFs’ cell cycle we observed in the present study may be a direct consequence of the DNA-damaging activity of HEMA/Bis-GMA in a prolonged, 24-h exposure. Also, modulation of these changes by the ascorbate or melatonin likely follow from the protective anti-oxidative actions of these substance against DNA damage induced by the model adhesive. Several molecular pathways link methacrylate-induced DNA damage and cell cycle perturbations, which creates also several opportunities for ascorbate or melatonin to exert their protective actions. TEGDMA in the millimolar range was reported to delay the HGFs cell cycle at the G2/M checkpoint associated with an early transient dephosphyralotion of ERK 1/2 and JNK and a late activation of the p53–p21(WAF-1)-pRb molecular pathway [38].

We show that the antioxidants ascorbate or melatonin exerts a protective effects against the apoptotic action of HEMA/Bis-GMA. We previously found a pro-apoptotic action of dental methacrylates. Both vitamin C and melatonin may also display pro-apoptotic effects, especially in cancer cells [39–42]. The mechanisms underlying apoptotic effects of these substances in cancer cells is not completely known. The question whether the mechanism may contain DSBs induced by methacrylates should be answered negatively, at least in the range of concentration which was applied in the present experiment. Urcan et al. [13] estimated that Bis-GMA at 0.09 mM induced 5 γ-H2AX foci per cell. This is too few to induce apoptosis, since the estimated rate of production of endogenous DSBs in humans is 50 per cell cycle [43]. Another question is whether the DSBs we observed in the present study might be a consequence of methacrylate-induced apoptosis? The answer to this question is presumably no. We did not observe comets typical for apoptosis in the present experiment. Such comets have no or very small head with virtually all the DNA in their tails [21]. Therefore, the mechanism underlying the induction of apoptosis by dental methacrylates is yet to be elucidated.

In summary, we showed in an in vitro study that the genotoxicity of a model dental adhesive HEMA/Bis-GMA included the induction of DSBs and interference with their repair. DSBs induced by HEMA/Bis-GMA may have, at least in part an oxidative character with vitamin C and melatonin acting as protective agents against DSBs, cell cycle changes and apoptosis induced by the adhesive. These substances may exert the in protective action because of their antioxidative properties. Therefore, vitamin C and melatonin can be considered as agents to reduce detrimental genotoxic effects of methacrylate-based dental filling.

This is interesting since, in the US, many individuals use sublingual melatonin tablets before bedtime which greatly increases the melatonin concentration in the oral cavity. This could, over time, reduce DSBs and oral cancer. Therefore, if one has methacrylate fillings perhaps they should use sublingual melatonin nightly.
